# Current News

**Published:** 1899-09

**Authors:** 


### Current News.

### THE NATIONAL ASSOCIATION OF DENTAL FACULTIES AND THE NATIONAL ASSOCIATION OF DENTAL EXAMINERS.

The members of the dental profession will be glad to learn that the difference existing for some years between the National Association of Dental Faculties and the National Association of Dental Examiners have been reconciled. These differences have been the cause of much friction between the two bodies.

The cause of the trouble was the refusal of the colleges to accept, and the adherence of the latter body to, various rules which have been crystallized into what is known as Rule 8 of its Code of Rules, Sections 1 and 2.

The attempted enforcement of this rule recently led to litigation in the State of Wisconsin. The State Board of Dental Examiners of that State refused to admit to registration the diplomas of the Chicago College of Dental Surgery, the Northwestern University Dental School, the Pennsylvania College of Dental Surgery, the Ohio Medical University Dental Department, the Philadelphia Dental College, and others, on the ground that they did not, in their preliminary examination, come up to that standard established by Rule 8, and demanded that graduates of these institutions presenting diplomas for registration should submit to examination by the Board as to their qualifications to practise dentistry.

This contention of the Board was resisted by a graduate of the Chicago College of Dental Surgery, who brought mandamus proceedings to compel the Board to accept his diploma. The Board moved to quash the proceedings, which motion was denied by the court with leave to the Board to file its answer. This was filed, and the case was in that condition at the time of the meeting of the two Associations at Niagara Falls on the 28th of July, 1899.

With a view to the adjustment of the difficulty, committees of conference were appointed by the two bodies, which, after going over the matters in dispute, agreed, on the side of the National Association of Dental Examiners, to recommend that Rule 8 be rescinded and that all colleges having membership in the National Association of Dental Faculties be placed upon the list of recognized schools, and that all litigation be withdrawn; and, on the side of the National Association of Dental Faculties, that a new rule governing the preliminary requirements for admission to the college courses should be adopted.

This action was ratified by the Association. The Examiners' Association adopted a new Rule 8, Sections 1 and 2 of which read as below, the remainder of the rule being substantially as before:

"Sec. 1. Colleges desiring recommendation to the State Board by the National Association of Dental Examiners shall make application for such recommendation through the Committee on Colleges, on blanks provided for that purpose. This rule to apply only to schools making application to the National Association of Dental Examiners for recommendation and such schools as may be dropped.

"Sec. 2. The following preliminary examination shall be required of students seeking admission to colleges recommended by this Association. The minimum preliminary educational requirements of colleges of this Association for the session of 1900 and 1901 shall be a certificate of entrance into the second year of a high school or its equivalent, the preliminary examination to be placed in the hands of the State Superintendent of Public Instruction, as adopted by the Missouri State Board."

The Faculties Association adopted the following rule governing the preliminary educational requirements of students:

"The minimum preliminary educational requirements of colleges of this Association, for the session of 1900 and 1901 shall be a certificate of entrance into the second year of a high school, or its equivalent, the preliminary examination to be placed in the hands of the State Superintendent of Public Instruction.

"Nothing in this rule shall be construed to interfere with colleges of this Association that are able to maintain a higher standard of preliminary education."

The cause of friction being removed, the disputes which have arisen, there is every assurance, will be speedily adjusted and the two bodies will thereafter work in harmony.

---

### NATIONAL DENTAL ASSOCIATION.

The next annual meeting of the National Dental Association will be held at Old Point Comfort, Va., beginning June 26, 1900. The date was selected to accommodate the large number of dentists who expect to attend the International Dental Congress, Paris, later in the season.

The election of officers resulted as follows:

President, Dr. B. Holly Smith, Baltimore, Md.; Vice-President for the East, Dr. John I. Hart, New York; Vice-President for the West, Dr. T. W. Brophy, Chicago; Vice-President for the South, Dr. M. F. Finley, Washington, D. C.; Recording Secretary, Geo. H. Cushing, Burbank, Cal.; Corresponding Secretary, Emma Eames Chase, St. Louis, Mo.; Treasurer, Henry W. Morgan, Nashville, Tenn.

*Executive Committee.*—H. A. Smith, Cincinnati; J. D. Patterson, Kansas City, Mo.; T. S. Waters, Baltimore, Md.

*Executive Council.*—H. J. Burkhart, Batavia, N. Y.; T. Fillebrown, Boston, Mass.; I. S. Cassidy, Covington, Ky.; W. E. Griswold, Denver, Col.; I. Y. Crawford, Nashville, Tenn.

---

### SOUTH DAKOTA STATE DENTAL SOCIETY.

The Seventeenth Annual Meeting of the South Dakota State Dental Society was held in Yankton, June 7, 8, and 9, 1899. The subject of crown- and bridge-work was very ably presented in a paper, and later demonstrated, by Dr. Gasler, of Chicago.

The officers elected for the ensuing year were W. O. Robinson, of Parker, President; W. W. Price, of Centreville, Vice-President; C. L. Blunt, of Yankton, Secretary and Treasurer; C. W. Stutenroth, of Watertown, Librarian.

Lead was selected as the place for the next meeting, and the time for holding the same was left to the Executive Committee.

C. L. Blunt,
*Secretary.*

---

### COLORADO STATE DENTAL ASSOCIATION.

At the Thirteenth Annual Meeting of the Colorado State Dental Association, held in Denver, June 13, 14, and 15, 1899, the following officers were elected:

President, A. C. Watson, Denver; First Vice-President, J. N. Chipley, Pueblo; Second Vice-President, Mary A. Bradner, Denver; Corresponding Secretary, Florence S. Green, Denver; Recording Secretary, L. S. Gilbert, Denver; Treasurer, William Smedley, Denver.

Florence S. Green,
*Corresponding Secretary.*

