# Correction

**Published:** 1899-11

**Authors:** 


### CORRECTION.

In the September number, in a notice of South Dakota State Dental Society, the name of the essayist should read Dr. H. J. Goslee, of Chicago, not Gasler, as printed.

